# Time between Collection and Storage Significantly Influences Bacterial Sequence Composition in Sputum Samples from Cystic Fibrosis Respiratory Infections

**DOI:** 10.1128/JCM.00764-14

**Published:** 2014-08

**Authors:** Leah Cuthbertson, Geraint B. Rogers, Alan W. Walker, Anna Oliver, Tarana Hafiz, Lucas R. Hoffman, Mary P. Carroll, Julian Parkhill, Kenneth D. Bruce, Christopher J. van der Gast

**Affiliations:** a NERC Centre for Ecology & Hydrology, Wallingford, United Kingdom; b Institute of Pharmaceutical Science, Molecular Microbiology Research Laboratory, King's College London, London, United Kingdom; c Wellcome Trust Sanger Institute, Hinxton, Cambridgeshire, United Kingdom; d Cystic Fibrosis Unit, Southampton University Hospitals, NHS Trust, Southampton, United Kingdom; e Department of Pediatrics, University of Washington, Seattle, Washington, USA; f Department of Microbiology, University of Washington, Seattle, Washington, USA; g SAHMRI Infection and Immunity Theme, School of Medicine, Flinders University, Bedford Park, Adelaide, Australia

## Abstract

Spontaneously expectorated sputum is traditionally used as the sampling method for the investigation of lower airway infections. While guidelines exist for the handling of these samples for culture-based diagnostic microbiology, there is no comparable consensus on their handling prior to culture-independent analysis. The increasing incorporation of culture-independent approaches in diagnostic microbiology means that it is of critical importance to assess potential biases. The aim of this study was to assess the impact of delayed freezing on culture-independent microbiological analyses and to identify acceptable parameters for sample handling. Sputum samples from eight adult cystic fibrosis (CF) patients were collected and aliquoted into sterile Bijou bottles. Aliquots were stored at room temperature before being frozen at −80°C for increasing intervals, up to a 72-h period. Samples were treated with propidium monoazide to distinguish live from dead cells prior to DNA extraction, and 16S rRNA gene pyrosequencing was used to characterize their bacterial compositions. Substantial variation was observed in samples with high-diversity bacterial communities over time, whereas little variation was observed in low-diversity communities dominated by recognized CF pathogens, regardless of time to freezing. Partitioning into common and rare species demonstrated that the rare species drove changes in similarity. The percentage abundance of anaerobes over the study significantly decreased after 12 h at room temperature (*P* = 0.008). Failure to stabilize samples at −80°C within 12 h of collection results in significant changes in the detected community composition.

## INTRODUCTION

Next-generation sequencing techniques are increasingly being used to characterize respiratory microbiota, including cystic fibrosis (CF) lower airway microbiota, in many lung diseases (see, e.g., references 1 to 4). These analyses have revealed microbial communities within the CF lung to be more complex and diverse than previously considered. Importantly, they have also detected many bacterial species that would not be reported by standard diagnostic microbiology techniques (see, e.g., references 5 and 6), as well as identified relationships between microbiota characteristics and host age, lung function, and disease progression (7–9).

In the majority of cases, these investigations relied on spontaneously expectorated sputum as a means of sampling the bacterial communities in the lower airways. Sputum is favored due to its ease of collection and the fact that culture-based microbiological studies of adult patients have traditionally used sputum samples as a basis for microbiological analysis. While guidelines exist for the handling of respiratory samples for culture-based diagnostic microbiology (10), there is no consensus on how such samples should be handled to ensure that culture-independent analyses yield results reflecting the microbes therein. With the increasing move toward the incorporation of culture-independent methods into diagnostic microbiology (11), it is increasingly important to identify and minimize areas of potential bias.

Postcollection sample transportation and storage represent periods during which changes can occur in bacterial communities of clinical samples, resulting in analytical bias due to, for example, bacterial proliferation, cell death, or degradation of nucleic acids. In order to minimize these biases, sputum samples collected for culture-independent analyses are typically stored at −80°C. However, many clinical sites, including those that treat cystic fibrosis (CF) patients, lack ready access to ultralow-temperature freezers, the standard recognized means of maintaining sample integrity and biobanking. As a result, sputum samples may remain at room temperature for extended periods, impacting both traditional and culture-independent analyses.

A prior study used 16S rRNA gene pyrosequencing of a single sample to assess the effect of extended periods of incubation at room temperature on bacterial community profiles but did not find significant divergence in community compositions over the study period (12). Conversely, in an earlier study using ribosomal transcripts to examine the V3 region of the 16S rRNA gene by quantitative PCR and denaturing gradient gel electrophoresis (DGGE), significant divergence in bacterial quantitation and community profiling was observed (13). RNA-based approaches have the advantage of limiting analysis to active cells. However, a related exclusion of nonviable cells and extracellular DNA can be achieved in DNA-based analysis through the treatment of samples with propidium monoazide (PMA), as we have demonstrated in previous analyses of CF sputum (14–16).

We hypothesized that the period of time between sample collection and stabilization by freezing is significantly related to the resultant bacterial community composition, as determined by 16S rRNA gene pyrosequencing in combination with PMA treatment. From this, our overarching aim was to determine an appropriate window of time from sample collection to storage at −80°C that would allow reliable culture-independent microbiological analysis of sputum samples.

## MATERIALS AND METHODS

### Sample collection.

Sputum samples were collected, under the full ethical approval of the Southampton and South West Hampshire Research Ethics Committee (protocol 06/Q1704/26), from eight adult patients attending the regional Cystic Fibrosis Centre in Southampton General Hospital for treatment for clinical exacerbation. Patients were chosen for their ability to provide sputum samples of 3 ml or more.

Samples were collected during physiotherapy, immediately aliquoted into sterile 5-ml Bijou bottles, and stored at room temperature until being frozen to −80°C at specified intervals. Samples at time zero (*t* = 0) were stored at −80°C immediately. The remaining samples were held at room temperature before storage at −80°C for 1, 3, 6, 9, 12, 18, 24, 36, 48, 60, and 72 h. The 72-h storage period was chosen to allow investigation of changes in the bacterial communities beyond the maximum 48-h storage recommended by Health Protection England for culture-based diagnostic microbiology (10).

### DNA extraction and pyrosequencing.

Sputum samples were washed three times with 1× phosphate-buffered saline. Free DNA and DNA from nonviable cells were excluded from analysis via cross-linking with PMA (14, 15) prior to DNA extraction, as previously described (16). Bacterial Golay barcode FLX amplicon pyrosequencing was performed using the primers 338F (5′-ACTCCTACGGGAGGCAGCAG) and 926R (5′-CCGTCAATTCMTTTRAGT). Initial generation of 16S rRNA gene amplicons involved a one-step PCR of 25 cycles using high-fidelity AccuPrime *Taq* DNA polymerase (Invitrogen, Carlsbad, CA). 454 pyrosequencing using the Lib-L kit was performed at the Wellcome Trust Sanger Institute, Hinxton, United Kingdom.

### Sequence analysis.

The mothur sequencing analysis platform was used to analyze the resulting data (17). Failed sequence reads, low-quality sequence ends, tags, and primers were initially removed, as were sequences below 400 bp and any sequences that included ambiguous base calls and homopolymers longer than 8 bases. Chimeras were removed in mothur using the Perseus software program (18). Sequences were assembled into operational taxonomic unit (OTU) clusters at 97% identity to give an approximation of species (19) and identified using the RDP reference database. Representative sequences were used to give appropriate species-level identifications for OTUs using the NCBI's BLASTN program.

### Statistical analysis.

All statistical analysis was performed in R (20). Three complementary measures of diversity were used as previously described (9) to identify changes in bacterial diversity in the same sample kept at room temperature for different durations: species richness (S*), the Shannon-Wiener diversity index (*H*′), and Simpson's index of diversity (1 − *D*) (21). The Bray-Curtis similarity index (*S*_BC_) (21) was calculated using randomized resampling to compare changes in community compositions over time at room temperature. To avoid potential biases due to sampling depth, randomized resampling with a uniform resample size (*n* = 200 to match the smallest sample size) (22) was carried out as described previously (9, 23). One thousand iterations of each resampling were performed to obtain the mean diversity and similarity coefficients and standard deviations of the means. The Berger-Parker (*d*) measure of dominance was calculated using the BiodiversityR package (24).

Bacterial species at *t* = 0 for each patient were partitioned into common and rare species using the inflection point method from rank abundance curves as previously described (25). A one-way analysis of variance (ANOVA) was calculated with two independent categorical variables, time and partition (common or rare), to compare the differences in similarities between common and rare species (20). The *post hoc* Tukey honestly significant difference (HSD) test was used in conjunction with ANOVA to compare treatment means in order to find significant differences (20).

Change in anaerobe abundance over time was investigated using nlme (26), and to fit data for mixed-effect models, *r*^*2*^ values were calculated using the MuMIn package (27).

The raw sequence data reported in this paper have been deposited in the NCBI Sequence Read Archive database (accession number SRP036061). A table listing the barcodes used is included as Table S2 in the supplemental material.

## RESULTS

Sputum samples were collected from eight patients and then aliquoted into 12 equal portions and stored at room temperature (mean ± standard error of mean [SE], 20.1°C ± 0.1°C) for specified intervals over a 72-h study period. Of the 96 sample aliquots sequenced, 12 were excluded from further analysis due to insufficient numbers of sequences (i.e., fewer than 200 sequences). A total of 182,989 bacterial sequences (mean ± SE, 2,178 ± 250 sequences/sample) were generated from 84 samples, which identified 51 genera and 78 distinct OTUs classified to the species level (see Table S1 in the supplemental material).

### Bacterial diversity.

Changes in bacterial diversity were assessed over the study period using recognized measures of diversity, namely, species richness (S*, the total number of species), the Shannon-Wiener index (*H*′, a metric accounting for both the number and the relative abundances of species), and Simpson's diversity index (1 − *D*, a measure of the probability that two species randomly selected from a sample will differ). S*, H′, and 1 − *D* were calculated for each sample from each patient over the 72-h study period, using randomized resampling as previously described (23), as pairwise comparisons are affected by large differences in sample size (28).

**FIG 1:**
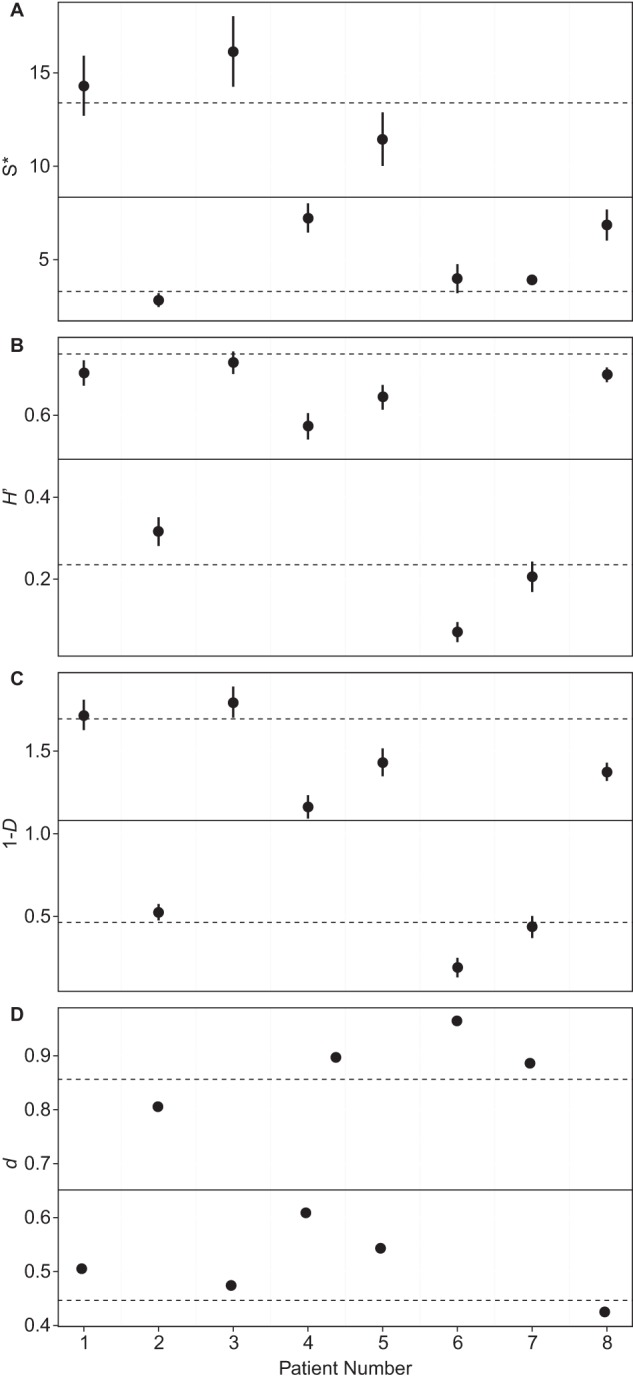
Comparison of the levels of diversity and dominance of bacterial communities across patients at *t* = 0. Values for species richness (S*) (A), the Shannon-Wiener index of diversity (*H*′) (B), and Simpson's index of diversity (1 − *D*) (C) are shown. The three diversity indices were calculated with a uniform resample size following 1,000 iterations in each instance. Error bars represent the standard deviations of the means (*n* = 1,000). In each instance, the overall mean (solid line) and the standard deviation of the mean (dashed lines) across patients are shown. (D) Also given are results for the Berger-Parker index of dominance (*d*).

High levels of variation were observed when we examined diversity measures, both between and within patients. In order to investigate this variation, sample diversity at *t* = 0 was plotted for S*, H′, and 1 − *D* (Fig. 1).

Typically, low-diversity communities are dominated by a few highly abundant species, whereas high-diversity communities are characterized by species that are more evenly distributed in their abundances (29). In order to explain the observed variation in diversity in the current study, the Berger-Parker index of dominance (*d*, the proportional abundance of the most abundant organism) was calculated for all *t* = 0 samples (Fig. 1) and for samples at all time points (see Fig. S1 in the supplemental material). Here, we also observed that low diversity (as defined by S*, *H*′, and 1 − *D*) was related to high dominance (*d*) in sample communities; for example, the *t* = 0 community of patient 6 was dominated by Pseudomonas aeruginosa (S* = 4, *d* = 0.96), and the *t* = 0 communities of patients 2 and 7 were dominated by Achromobacter xylosoxidans (for patient 2, S* = 3 and *d* = 0.88; for patient 7, S* = 4 and *d* = 0.80), while patient 1 showed a much more diverse *t* = 0 community (S* = 14, *d* = 0.51) (Fig. 1 and S1). Given the high level of variation in diversity between patients, linked to species dominance, these measures were unsuitable as metrics to indicate changes over time, suggesting that comparisons of community similarities over time would be more appropriate.

### Bacterial community membership.

The Bray-Curtis measure of similarity (*S*_BC_, which accounts for the number and abundance of species present in each community and those that are shared) was used to compare changes in community composition between samples, resulting in a value between 0 and 1 (higher values indicate greater similarity). As for the diversity measures, community compositions were compared using the sample at *t* = 0 and each subsequent sample. Using randomized resampling, total change in *S*_BC_ similarity was assessed (22).

For both PCR and sequencing, sampling bias can result in variation between repeat samples of the same community (12). In order to evaluate whether changes in similarity across the sampling period were due to true community changes or within-sample variation, a cutoff value for similarity was calculated using eight samples, each sequenced in triplicate. The overall mean *S*_BC_ similarity between sample replicates was 0.782 ± 0.1 (mean ± SE) (*n* = 24); therefore, similarity values below 0.682 (0.782 ± 0.10) between *t* = 0 and subsequent samples were judged to be different from the original sample.

**FIG 2:**
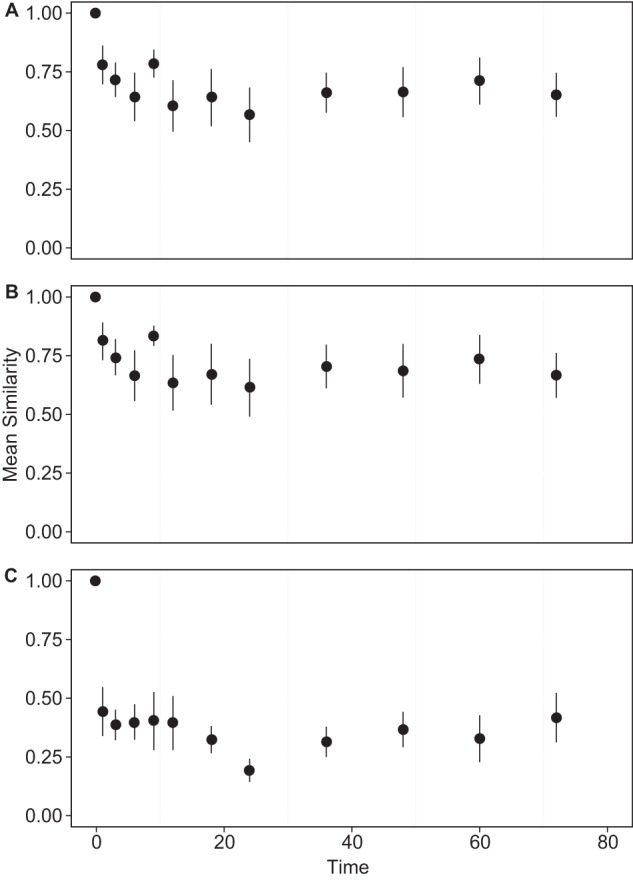
Mean changes in bacterial community composition from *t* = 0 across patients over time using the Bray-Curtis index of similarity for whole communities (A), common species (B), and rare species (C). Error bars represent the standard deviations of the means (*n* = 8).

**FIG 3:**
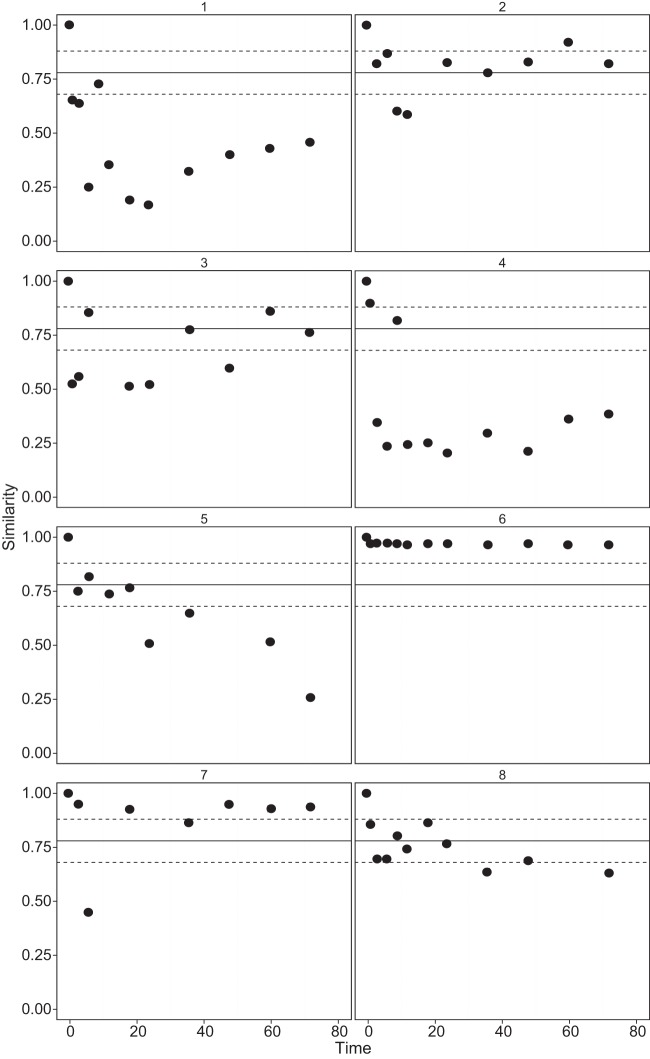
Changes in community composition from *t* = 0 for each patient over time according to the Bray-Curtis index of similarity. Solid lines represent the overall mean similarity from within-sample replicates, and dashed lines represent the standard errors of the means (*n* = 24).

The mean change in similarity over the study period was not judged to be significant when we accounted for within-sample variation (Fig. 2A). When diversity measures were examined for individual patients, high levels of variation were observed (Fig. 3). These results were compared to the value of dominance calculated previously using *d* (Fig. 1). The greater the relative abundance of the dominant species, the lower the variation in community similarity over the study period (*P* = 0.03, *r*^*2*^ = 0.05). This finding indicated that samples more highly dominated by a single species and, hence, having low overall diversity were less likely to show a significant change in community similarity with longer times at room temperature prior to being frozen.

To further examine how dominant species affect community similarity, rank abundance curves were used to partition sample communities at *t* = 0 into common and rare species groups (see Fig. S2 in the supplemental material) (25). Change in *S*_BC_ similarity was calculated from *t* = 0 samples for the partitioned groups, revealing much greater variation in species characterized as rare (Fig. 2). By ANOVA, a significant difference in similarity was observed between the common and rare species (*F*_1,148_ = 77.93, *P* < 0.001). *Post hoc* Tukey HSD testing revealed that the difference in similarity between *t* = 0 species and the rare species were on average 30.93% lower than the differences observed in the common species.

### Differential impact on aerobic and anaerobic species.

**FIG 4:**
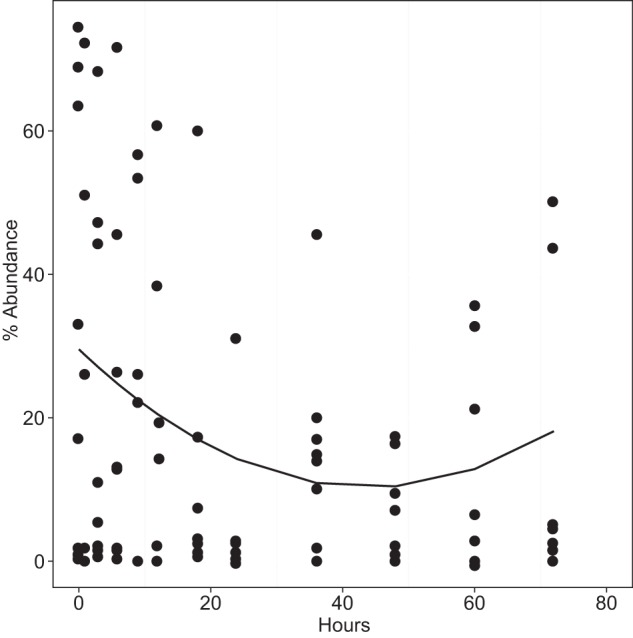
Changes in anaerobic species abundance for all patients over time. Circles represent percentages of abundance of anaerobic species for each patient at a given time point. A second-order polynomial model has been fitted to the data (*r*^2^ = 0.08, *P* = 0.004).

Over the study period, samples were aliquoted and stored in sterile sample containers; it was expected that this would result in a decrease in anaerobic species due to prolonged exposure to atmospheric oxygen, resulting in preferential conditions for aerobic populations. To that end, bacteria were partitioned into aerobic/facultative anaerobes and strict anaerobic species. A mixed-effect model was used to investigate the change in percentage abundance of anaerobic species present in each sample over the study period. The best-fit distribution was a second-order polynomial relationship (*r*^*2*^ = 0.08, *P* = 0.004) (Fig. 4). This distribution indicated a consistent decline in the relative abundance of anaerobic species over the first 48 h, followed by an increase during the following 24 h. Using mixed-effect models, the decline in anaerobes was found to represent a significant divergence from anaerobe numbers in the original sample after 18 h (*P* = 0.008). This decline continued until after 48 h of storage at room temperature, at which point the percentage abundance of anaerobes started to increase. This finding suggests that changes in anaerobe abundance were due to sputum storage in sealed containers, which allowed anaerobic organisms to proliferate after available oxygen had been depleted, resulting in community divergence.

## DISCUSSION

The handling and storage of respiratory samples can substantially bias the results of microbiological analyses. The aim of the current study was to determine an acceptable period of time between sputum sample collection and storage prior to use of next-generation sequencing to characterize CF lower airway microbiota. Previous studies based on diagnostic bacterial culture suggest that 48 h can elapse from specimen collection to either freezing or processing and still provide comparable results (10). However, with the increasing incorporation of culture-independent approaches to diagnostic microbiology (11), it is vital to identify and mitigate all potential biases relevant for these more sensitive, culture-independent techniques.

Changes in bacterial diversity as a result of storage at room temperature for different intervals prior to freezing were assessed within samples from each patient. We found that diversity was highly variable across patients at *t* = 0 (Fig. 1) and within patients over storage time postcollection (see Fig. S1 in the supplemental material). This variability could be attributed to differences in dominance within sample communities. Freshly collected (*t* = 0) samples with low species diversity were found to be highly dominated by recognized CF pathogens; conversely, those samples with a more diverse community were not dominated by a particular bacterial species. Furthermore, communities dominated by fewer species changed less with increasing time at room temperature than those with higher diversities. Given the differences in diversity between and within patient samples, commonly used measures of diversity, e.g., species richness, Shannon-Wiener diversity index, and Simpson's diversity index, were unsuitable as metrics of change in the bacterial communities during storage.

Using the Bray-Curtis index of similarity, we assessed how community composition changed from that in the original (*t* = 0) sample. As previous studies have indicated that bacterial communities within the CF lungs are not homogenously distributed (30), this may result in a variation within different portions of a single sample (12). In order to account for within-sample variance, eight CF sputum samples were sequenced in triplicate, using different sample aliquots for each replicate, and the similarity between replicates was calculated. Then, we analyzed all samples for all subjects, finding that the shortest period of sample storage within which a change in similarity was observed, beyond that expected for within-sample variation, was 1 h after sputum collection (Fig. 2). In addition, where a community was dominated by a few or one species, the variation in community similarity was found to be significantly lower than in more-diverse communities. However, since it is difficult to guess the microbial diversity in a sputum sample *a priori*, our results suggest that sputum samples should be frozen within 1 h of collection in order to obtain the best possible representation of the true community when culture-independent analyses are used.

Previous studies have demonstrated the value of partitioning bacterial communities in respiratory infections into common and rare species groups (9). Categorization of component species provides useful insights into communities that would be neglected without such a distinction (3, 9). When samples were partitioned into common and rare species in the current study, a greater level of community stability with difference in storage was associated with species defined as common than with species defined as rare, suggesting that characterization of the latter group will be most affected by a delay in sample freezing. This effect accounted for the greater change in similarity observed in more-diverse communities that have a wide range of rare species present within the community (Fig. 2).

We hypothesized that prolonged exposure to atmospheric levels of oxygen would result in a decrease in the relative abundance of viable, strictly anaerobic species within the sputum samples. Despite high variability in anaerobe relative abundance between samples, a statistically significant second-order polynomial relationship was found between storage duration at room temperature and anaerobe abundance, with the latter decreasing for up to 48 h, followed by an increase after that time (Fig. 4). A potential explanation for this relationship is the reduction in oxygen tension as a result of the growth of aerobic and aerotolerant species in the sealed Bijou containers. Furthermore, the decline in anaerobe abundance, which represents a major shift in the community, was found to be significantly different from that of *t* = 0 samples after 12 h (Fig. 4). This effect can potentially lead to under- or overestimation of the importance of anaerobic species in disease progression, depending on the period elapsed between storage and freezing.

Sputum samples are one of the most widely used ways of sampling lower respiratory tract infections. Researchers are now moving toward incorporating culture-independent techniques to analyze the microbial determinant of these conditions and make informed treatment choices. In the current study, we found that the optimal window for sample storage at room temperature before freezing at −80°C is within 1 h of collection. In practical terms, it may not be possible to store a sample within 1 h of collection. In this event, our results indicate an acceptable window of up to 12 h without significant divergence in community composition. While this work has focused on CF airway infections, these findings are important for the analysis of microbiota from samples of patients with other respiratory conditions.

## Supplementary Material

Supplemental material
