# Supplementary material for: Time between Collection and Storage Significantly Influences Bacterial Sequence Composition in Sputum Samples from Cystic Fibrosis Respiratory Infections
Source: J Clin Microbiol. 2014 Aug;52(8):3011–6. doi: 10.1128/JCM.00764-14 (PMC4136140; doi:10.1128/JCM.00764-14)
Supplement: Supplemental material [file supp_52_8_3011__index.html]

Time between Collection and Storage Significantly Influences Bacterial Sequence Composition in Sputum Samples from Cystic Fibrosis Respiratory Infections — Supplemental material 

# Time between Collection and Storage Significantly Influences Bacterial Sequence Composition in Sputum Samples from Cystic Fibrosis Respiratory Infections

## Supplemental material

**Files in this Data Supplement:**

- Supplemental file 1 -

  Fig. S1 (Changes in diversity and dominance of bacterial communities within individual patients over time)

  PDF, 231K
- Supplemental file 2 -

  Fig. S2 (Rank abundance curves for each patient bacterial community at time zero)

  PDF, 190K
- Supplemental file 3 -

  Table S1 (Bacterial species identified in eight sputum samples collected from CF patients)

  PDF, 338K
- Supplemental file 4 -

  Table S2 (Barcodes for sequence data deposited in the NCBI Short Read Archive database)

  PDF, 109K
