# Supplementary material for: Time between Collection and Storage Significantly Influences Bacterial Sequence Composition in Sputum Samples from Cystic Fibrosis Respiratory Infections
Source: J Clin Microbiol. 2014 Aug;52(8):3011–6. doi: 10.1128/JCM.00764-14 (PMC4136140; doi:10.1128/JCM.00764-14)
Supplement: Supplemental material [file JCM.00764-14_zjm999093634so1.pdf]

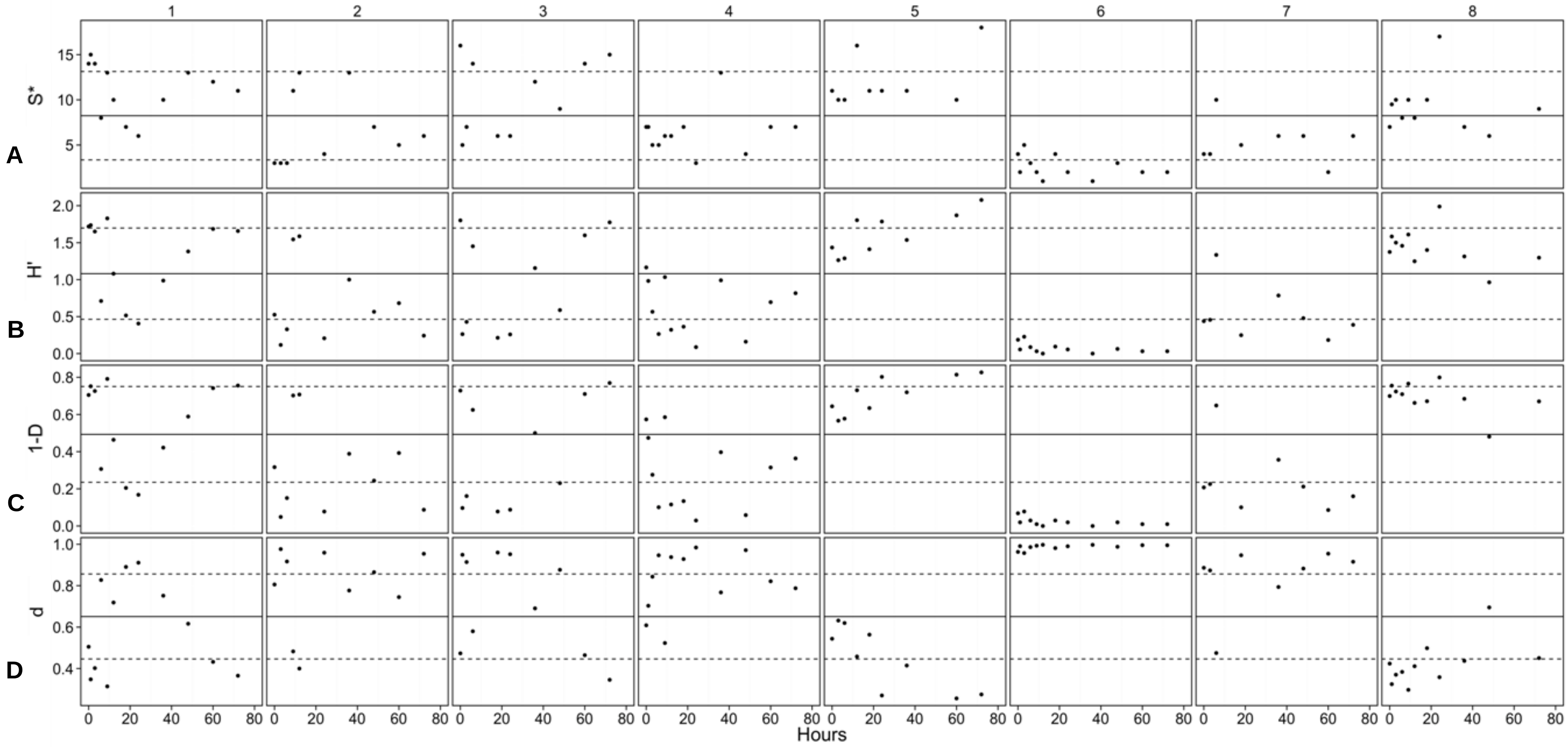

Fig. S1. Changes in diversity and dominance of bacterial communities within individual patients over time. Values of (A) species richness (*S**), (B) Shannon-Wiener index of diversity (*H*'), and (C) Simpson's index of diversity (1-*D*) are shown. The three diversity indices were calculated with a uniform re-sample size following 1000 iterations in each instance. Error bars represent the standard deviation of the mean (*n* = 1000). Also given is (D) the Berger-Parker index of dominance (*d*). In each instance, the overall mean (solid line) and the standard deviation of the mean (dashed lines) are shown.
