# Supplementary material for: Time between Collection and Storage Significantly Influences Bacterial Sequence Composition in Sputum Samples from Cystic Fibrosis Respiratory Infections
Source: J Clin Microbiol. 2014 Aug;52(8):3011–6. doi: 10.1128/JCM.00764-14 (PMC4136140; doi:10.1128/JCM.00764-14)
Supplement: Supplemental material [file JCM.00764-14_zjm999093634so2.pdf]

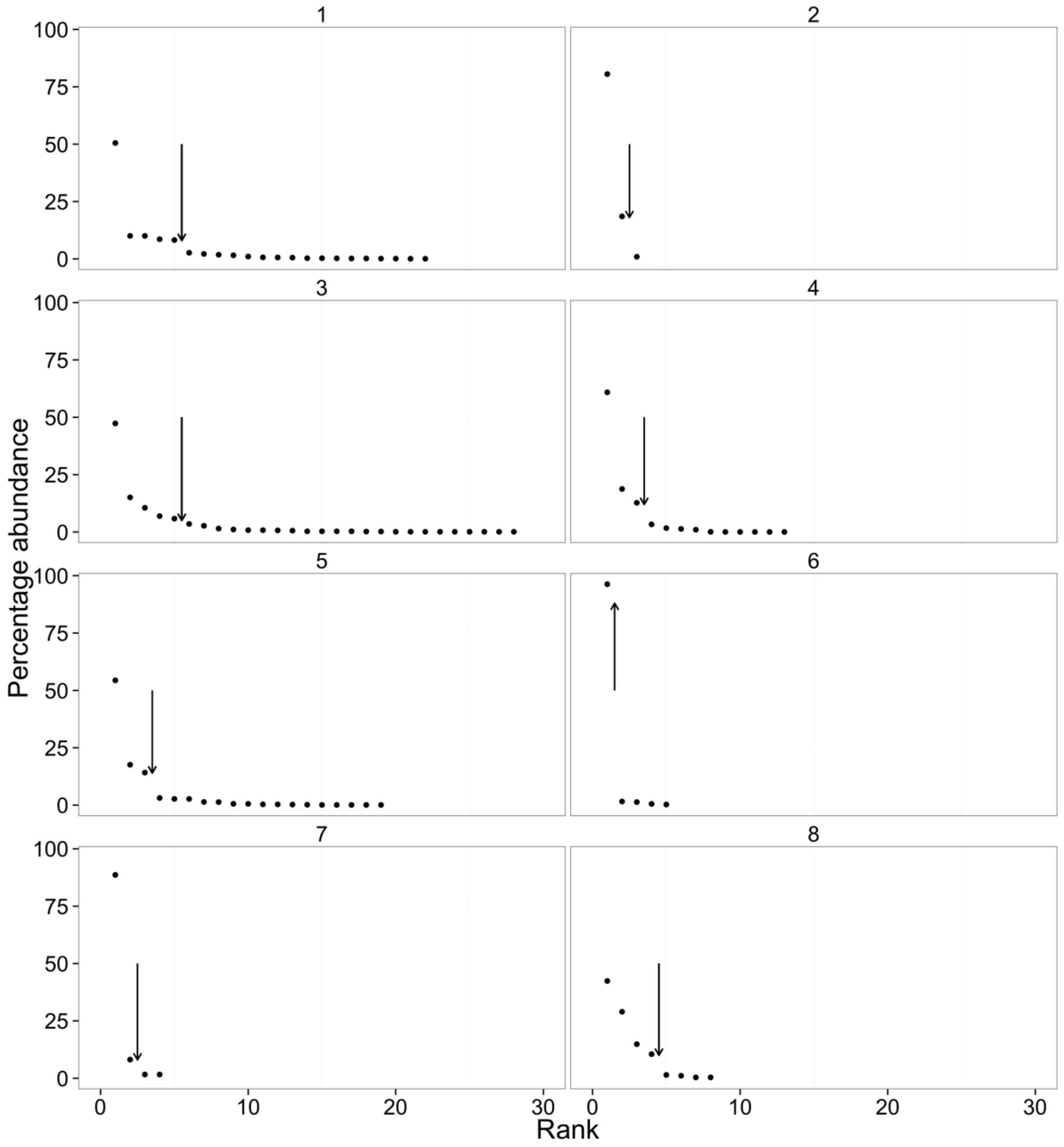

Fig. S2. Rank abundance curves for each patient bacterial community at t=0. Abundances are expressed as percentage of total abundance within each community. The arrows indicate the inflection point of each curve. Species to the left of the arrow were classified as common, and those at the right side were classified as rare.
