# Supplementary material for: Time between Collection and Storage Significantly Influences Bacterial Sequence Composition in Sputum Samples from Cystic Fibrosis Respiratory Infections
Source: J Clin Microbiol. 2014 Aug;52(8):3011–6. doi: 10.1128/JCM.00764-14 (PMC4136140; doi:10.1128/JCM.00764-14)
Supplement: Supplemental material [file JCM.00764-14_zjm999093634so3.pdf]

Table S1 Bacterial species identified from eight sputum samples collected from CF patients. Species-level identities of detected taxa are reported here. However, given the length of the ribosomal sequences analysed, these identities should be considered putative. Ae denotes aerobe and An, Anaerobe. Only strict anaerobes were classified as anaerobes, whereas aerobes, facultative anaerobes, and microaerophiles were classified as aerobes. The relative abundance of the bacterial species at t=0 and t=18 are provided.

				Patient 1		Patient 2		Patient 3		Patient 4		Patient 5		Patient 6		Patient 7		Patient 8	
Class	Family	Taxon name	Code	t=0	t=18	t=0	t=18	t=0	t=18	t=0	t=18	t=0	t=18	t=0	t=18	t=0	t=18	t=0	t=18
Actinobacteria	Actinomycetaceae	*Actinomyces odontolyticus*	Ae	0.6	0.13	0	0	0.3	0	3.33	0.69	2.7	3.18	0	0	0	0	0	0.33
		Actinomyces oris	Ae	0	0	0	0	0	0.01	0	0	0	0	0	0	0	0	0	0
		Actinobaculum schaalii	Ae	0	0	0	0	0	0	0	0	0	0	0	0	0	0	0	0
	Corynebacteriaceae	*Corynebacterium durum*	Ae	0	0.02	0	0	0	0	0	0	0	0	0	0	0	0	0	0
		Corynebacterium pseudogenitalium	Ae	0	0	0	0	0	0.01	0	0	0	0	0	0	0	0	0	0
	Micrococcaceae	*Rothia mucilaginosa*	Ae	0	0.02	0	0	0	0	0	0	0	0	0	0	0	0	0	0.33
	Propionibacteriaceae	*Propionibacterium propionicum*	Ae	0	0	0	0	0.1	0	0	0	0	0	0	0	0	0	0	0
	Bifidobacteriaceae	*Scardovia wiggsiae*	An	0	0	0	0	0	0	0	0	0	0	0	0	0	0	0	0
	Coriobacteriaceae	*Atopobium parvulum*	An	0.3	0.06	0	0	0	0.01	1.01	0.11	1.39	1.48	0	0	0	0	0	0
Bacteroidetes	Bacteroidaceae	*Bacteroides acidofaciens*	An	0.66	0.07	0	0	0.3	0	0	0	0	0	0	0	0	0	0	0
	Porphyromonadaceae	*Barnesiella intestinihominis*	An	0	0	0	0	0	0.01	0	0	0	0	0	0	0	0	0	0
		Porphyromonas catoniae	An	10	3.19	0.93	0	0.2	0	1.35	0.46	0.08	0	0	0.03	0	0	0	0
		Porphyromonas endodontalis	An	0	0	0	0	0	0.04	0.03	0	0.16	0	0	0	0	0	0	0
		Tannerella forsythia	An	0	0.02	0	0	0	0	0	0	0	0	0	0	0	0	0	0
	Prevotellaceae	*Prevotella denticola*	An	0	0	0	0	0	0	0	0	0	0	0	0	0	0	0	0
		Prevotella enoeca	An	0	0.02	0	0	6.93	0.53	0	0	0	0	0	0.03	0	0	0	0
		Prevotella loescheii	An	0	0	0	0	0	0	0	0.11	0	0	0	0.06	0	0	0	0
		Prevotella melaninogenica	An	50.5	3.15	0	0	3.52	0.2	60.9	1.89	54.4	56.4	0.26	0.25	1.63	0.28	1.45	0.99
		Prevotella nigrescens	An	1.57	0.24	0	0	2.71	0.12	0.03	0	0	0	0	0.06	0	0	0	0
		Prevotella oralis	An	0	0	0	0	0	0	0	0	0	0	0	0	0	0	0	0
		Prevotella oris	An	0.18	0.04	0	0	1.51	0.09	0.07	0	0	0	0	0	0	0	0	0
		Prevotella pallens	An	0	0	0	0	0	0.03	0	0	0	0	0	0	0	0	0	0

Class	Family	Taxon name	Code	Patient 1		Patient 2		Patient 3		Patient 4		Patient 5		Patient 6		Patient 7		Patient 8	
				t=0	t=18	t=0	t-18	t=0	t=18	t=0	t=18	t=0	t=18	t=0	t=18	t=0	t=18	t=0	t=18
		Prevotella pleuritidis	An	0	0	0	0	0	0	0	0	0	0.21	0	0	0	0	0	0
		Prevotella salivae	An	2.18	0.17	0	0	0.1	0.04	0.03	0	0.57	0	0	0.03	0	0	14.9	14.5
		Prevotella veroralis	An	0	0	0	0	0	0	0	0	0	0	0	0	0	0	0	0
		Prevotella maculosa	An	0.18	0.02	0	0	0	0	0	0	0	0	0	0	0	0	0	0
		Prevotella tannerae	An	0	0	0	0	0	0	0	0	0	0	0	0	0	0	0	0
	Rikenellaceae	*Alistipes finegoldii*	An	0	0	0	0	0	0	0	0	0	0	0	0	0	0	0	0
Flavobacteria	Flavobacteriaceae	*Capnocytophaga granulosa*	Ae	1.03	0.04	0	0	0	0	0	0.06	0	0	0	0	0	0	0	0
		Capnocytophaga ochracea	Ae	0	0	0	0	0	0	0	0	0	0	0	0	0	0	0	0
		Capnocytophaga sputigena	Ae	1.81	0.13	0	0	0	0	0	0.06	0	0	0	0	0	0.28	0	0
Bacilli	Bacillales Family XI	*Gemella morbillorum*	Ae	0	0	0	0	0.6	0.04	0	0.06	0	0	0	0	0	0	0	0
	Staphylococcaceae	*Staphylococcus aureus*	Ae,P	0	0	0	0	0	0	0	0	0.08	0	0	0	0	0	0	0.99
	Aerococcaceae	*Abiotrophia defectiva*	Ae	0	0	0	0	0.1	0	0	0	0.25	0.42	0	0	0	0	0	0
	Carnobacteriaceae(77)	*Granulicatella adiacens*	Ae	0.24	0.02	0	0	0.1	0.01	0	0	0.57	0.42	0	0	0	0	0	0
	Lactobacillaceae	*Lactobacillus acidophilus*	Ae	0	0	0	0	0	0	0	0	0.08	0	0	0	0	0	0	0
		Lactobacillus fermentum	Ae	0	0	0	0	0	0	0	0	2.7	10.4	0	0	0	0	0	0.33
		Lactobacillus johnsonii	Ae	0	0	0	0	0	0	0	0	0.08	0.42	0	0	0	0	0	0
		Lactobacillus oris	Ae	0	0	0	0	0	0	0	0	0.08	0.21	0	0	0	0	0	0
		Lactobacillus salivarius	Ae	0	0	0	0	0	0	0	0	0	0	0	0	0	0	1.09	0.33
		Lactobacillus casei	Ae	0	0	0	0	0	0	0.03	0	0.33	0.42	0	0	0	0	0	0
	Streptococcaceae	*Streptococcus mitis group*	Ae,P	10	2.32	0	0.45	10.6	1.13	12.7	1.89	14.2	16.7	1.59	0.37	1.63	0.28	42.4	49.8
Clostridia	Clostridiaceae	*Clostridium cocleatum*	An	0	0	0	0	0	0	0	0	0	0	0	0	0	0	0	0
	Clostridiales Family XI	*Finegoldia magna*	An	0	0	0	0	0	0	0	0	0	0	0	0	0	0	0	0
	Clostridiales Family XI	*Parvimonas micra*	An	0	0	0	0	0	0	0	0	0	0	0	0	0	0	0	0
	Clostridiales Family XIII	*Mogibacterium diversum*	An	0	0	0	0	0	0	0	0	0	0	0	0	0	0	0	0
	Eubacteriaceae	*Eubacterium brachy*	An	0	0	0	0	0.1	0	0	0	0	0	0	0	0	0	0	0
	Lachnospiraceae	*Oribacterium sinus*	An	0	0	0	0	0	0.01	0	0	0	0	0	0	0	0	0	0
		Stomatobaculum longum	An	0	0	0	0	0.2	0.01	0	0	0	0	0	0	0	0	0	0
		Lachnoanaerobaculum orale	An	0	0	0	0	0	0	0	0	0	0	0	0	0	0	0	0
	Peptococcaceae_1	*Peptococcus niger*	An	0	0	0	0	0	0	0	0	0	0	0	0	0	0	0	0
Negativicutes	Veillonellaceae	*Dialister pneumosintes*	An	0	0	0	0	0.1	0	0	0	0	0	0	0	0	0	0	0

				Patient 1		Patient 2		Patient 3		Patient 4		Patient 5		Patient 6		Patient 7		Patient 8	
**Class**	**Family**	**Taxon name**	**Code**	**t=0**	**t=18**	**t=0**	**t=18**	**t=0**	**t=18**	**t=0**	**t=18**	**t=0**	**t=18**	**t=0**	**t=18**	**t=0**	**t=18**	**t=0**	**t=18**
		Megasphaera micronuciformis	An	0.12	0	0	0	0.3	0.04	0	0.11	0	0.21	0	0	0	0.28	0	0.33
		Selenomonas noxia	An	0	0	0	0	0	0.02	0	0	0	0	0	0	0	0	0	0
		Veillonella parvula	An	2.66	0.44	0	0	0.8	0.02	0	0.17	17.6	1.69	0.53	0.12	0	0.28	0.36	1.32
Fusobacteria	Fusobacteriaceae	*Fusobacterium necrophorum*	An	0	0	0	0	0.3	0	0	0	0	0	0	0	0	0	0	0
		Fusobacterium nucleatum	An	0.12	0	0	0.91	15.1	1.09	0.03	0.11	0.33	0	0	0.47	0	0	0	0.33
		Leptotrichia buccalis	An	0.3	0.06	0	0	0	0	0	0	0	0	0	0	0	0	0	0
	Leptotrichiaceae	*Sneathis sanguinegens*	An	0	0	0	0	0.7	0.07	0	0	0	0	0	0	0	0.28	0.36	0
Betaproteobacteria	Alcaligenaceae	*Achromobacter xylosoxidans*	Ae,P	8.58	0.81	80.6	95.9	5.83	0.36	1.7	1.43	1.31	7.42	1.32	0.28	88.6	94.7	29	22.8
	Burkholderiaceae	*Burkholderia cepacia complex member*	Ae,P	0	0	0	0	1.11	0	0	0	0	0	0	0	0	0	0	0
		Ralstonia pickettii	Ae	0	0	0	0	0	0	0	0	0	0	0	0	0	0	0	0
	Comamonadaceae	*Comamonas testeroni*	Ae	0	0	0	0.45	0	0	0	0	0	0	0	0	0	0	0	0
	Burkholderiaceae	*Lautropia mirabilis*	Ae	0.06	0	0	0	0	0	0	0	0	0	0	0	0	0	0	0
	Neisseriaceae	*Eikenella corrodens*	Ae	0.06	0	0	0	0.8	0.1	0	0	0	0	0	0.06	0	0	0	0
		Kingella oralis	Ae	0	0	0	0	0.1	0	0	0	0	0	0	0	0	0	0	0
		Neisseria bacilliformis	Ae	0.54	0	0	0	0	0	0	0	0	0	0	0	0	0	0	0
Epsilonproteobacteria	Campylobacteraceae	*Campylobacter concisus*	Ae	0	0	0	0	0	0	0	0	0	0	0	0	0	0	0	0
Gammaproteobacteria	Enterobacteriaceae	*Escherichia coli*	Ae	0	0	0	0	0	0	0	0	0	0	0	0	0	0	0	0
	Pasteurellaceae	*Haemophilus influenzae*	Ae,P	0	0	0	0	0	0	0	0	0	0	0	0	0	0	0	0
		Haemophilus parainfluenzae	Ae	0	0	0	0	0.1	0	0	0	0	0	0	0	0	0	0	0
	Moraxellaceae	*Acinetobacter johnsonii*	Ae	0	0	0	0	0	0	0	0	0	0	0	0	0	0	0	0
	Pseudomonadaceae	*Pseudomonas aeruginosa*	Ae,P	8.22	89	18.5	2.27	47.3	96	18.8	92.8	3.11	0.42	96.3	98.1	8.13	3.64	10.5	7.26
		Pseudomonas fluorescens	Ae	0	0	0	0	0	0	0	0	0	0	0	0.03	0	0	0	0
		Pseudomonas putida	Ae	0	0	0	0	0	0	0	0	0	0	0	0	0	0	0	0.33
		Pseudomonas mendocina	Ae	0	0	0	0	0.1	0	0	0	0	0	0	0	0	0	0	0
	Xanthomonadaceae	*Stenotrophomonas maltophillia*	Ae,P	0	0	0	0	0	0	0	0	0	0	0	0.06	0	0	0	0
Mollicutes	Mycoplasmataceae	*Mycoplasma saliverium*	Ae	0	0	0	0	0	0	0	0	0	0	0	0	0	0	0	0
