# Supplementary material for: Time between Collection and Storage Significantly Influences Bacterial Sequence Composition in Sputum Samples from Cystic Fibrosis Respiratory Infections
Source: J Clin Microbiol. 2014 Aug;52(8):3011–6. doi: 10.1128/JCM.00764-14 (PMC4136140; doi:10.1128/JCM.00764-14)
Supplement: Supplemental material [file JCM.00764-14_zjm999093634so4.pdf]

Table S2. The raw sequence data reported in this paper have been deposited in the NCBI Short Read Archive database (Accession number. SRP036061). A list of barcodes used and their associated sample are listed below.

Barcode	Patient	Hour
AGATGTTCTGCT	**1**	0
AGCTATCCACGA	**1**	1
AGTGCGATGCGT	**1**	3
ATCTGAGCTGGT	**1**	6
AGTTAGTGCGTC	**1**	9
ATACAGAGCTCC	**1**	12
AGAACACGTCTC	**1**	18
ATCCGATCACAG	**1**	24
ATCTACTACACG	**1**	36
AGCTCCATACAG	**1**	48
ATCACGTAGCGG	**1**	60
ATCTGGTGCTAT	**1**	72
ATATGCCAGTGC	**2**	0
ATCCTCAGTAGT	**2**	3
ATACACGTGGCG	**2**	6
ATCTCTGGCATA	**2**	9
ATCGTACAACTC	**2**	12
ATCAGGCGTGTG	**2**	24
ATGGATACGCTC	**2**	36
ATGGCAGCTCTA	**2**	48
ATGCACTGGCGA	**2**	60
ATGCAGCTCAGT	**2**	72
ACCACATACATC	**3**	0
ACATGTCACGTG	**3**	1
ACCTGTCTCTCT	**3**	3
ACTGATCCTAGT	**3**	6
ACGAGTGCTATC	**3**	18
ACGCTCATGGAT	**3**	24

Barcode	Patient	Hour
AGACCGTCAGAC	**3**	36
ACAGTTGCGCGA	**3**	48
ACACTGTTCATG	**3**	60
ACAGCTAGCTTG	**3**	72
ACTACGTGTGGT	**4**	0
AGTCTCGCATAT	**4**	1
AGACGTGCACTG	**4**	3
ATAATCTCGTCG	**4**	6
ACGCAACTGCTA	**4**	9
AGCTTGACAGCT	**4**	12
AGGTGTGATCGC	**4**	18
AGAGAGCAAGTG	**4**	24
AGCGCTGATGTG	**4**	36
AGAGCAAGAGCA	**4**	48
ACGTGAGAGAAT	**4**	60
AGTGTTCGATCG	**4**	72
AGTACGCTCGAG	**6**	0
ATGAGACTCCAC	**6**	3
AGTGTCACGGTG	**6**	6
AGTGAGAGAAGC	**6**	12
ATATCGCTACTG	**6**	18
ATCGCGGACGAT	**6**	24
ATAGGCGATCTC	**6**	36
ATGCCTGAGCAG	**6**	60
ATGACTCATTCG	**6**	72
ACGGATCGTCAG	**7**	0
ACGCTATCTGGA	**7**	1
AGTACTGCAGGC	**7**	3

Barcode	Patient	Hour
AGATCGGCTCGA	**7**	6
AGCACACCTACA	**7**	9
ACGTCTGTAGCA	**7**	12
AGCGAGCTATCT	**7**	18
ACTCACGGTATG	**7**	24
AGCAGCACTTGT	**7**	36
AGTCTACTCTGA	**7**	48
ACTGTGACTTCA	**7**	60
ACTCAGATACTC	**7**	72
ACGTACTCAGTG	**9**	0
AGCACGAGCCTA	**9**	3
AGCCATACTGAC	**9**	6
ACTAGCTCCATA	**9**	18
ACGCGCAGATAC	**9**	36
ACTTGTAGCAGC	**9**	48
ATCGATCTGTGG	**9**	60
ACGTTAGCACAC	**9**	72
ACACTAGATCCG	**11**	0
ACAGAGTCGGCT	**11**	1
ACGCGATACTGG	**11**	3
ACTGACAGCCAT	**11**	6
ACCGCAGAGTCA	**11**	9
ACAGCAGTGGTC	**11**	12
ACCAGACGATGC	**11**	18
ACCTCGATCAGA	**11**	24
ACGGTGAGTGTC	**11**	36
ACCAGCGACTAG	**11**	48
ACAGACCACTCA	**11**	72
